# Can moving in a redundant workspace accelerate motor adaptation?

**DOI:** 10.1152/jn.00458.2021

**Published:** 2022-11-23

**Authors:** Jahangir Esfandiari, Seyedsina Razavizadeh, Max-Philipp Stenner

**Affiliations:** ^1^Department of Neurology, Otto-von-Guericke University, Magdeburg, Germany; ^2^Department of Behavioral Neurology, Leibniz Institute for Neurobiology, Magdeburg, Germany; ^3^Center for Behavioral Brain Sciences, Magdeburg, Germany

**Keywords:** error-based learning, motor adaptation, movement variability, reaching, workspace redundancy

## Abstract

Variability in behavior can be a manifestation of unwanted noise. However, variability can also reflect exploration and benefit learning. For example, it has been shown that interindividual differences in motor learning can be partly explained by differences in movement variability at baseline. Here, we examined whether permitting versus constraining movement variability via target shape alters motor learning rate in one and the same individual. Healthy young subjects made reaching movements to visual targets in two-dimensional space with their unseen hand. During an initial priming phase, the shape of targets allowed for movement variability either in direction (arc-shaped targets), or, in a separate session, in extent (radially oriented line-shaped targets), while requiring highly precise movements in the other spatial dimension, respectively. In subsequent test phases in each session, we quantified the rate of (single-trial) motor adaptation to visuomotor perturbations along these two spatial dimensions (rotation and gain). During priming, we observed higher variability in movement direction for arc-shaped targets, compared with radial line-shaped targets, and vice versa for variability in movement extent. As predicted, participants adapted more to a visuomotor rotation following priming with arc-shaped targets, compared with radial line-shaped targets, and vice versa for adaptation to a change in visuomotor gain. This effect was prominent in the part of the examined workspace where variability in initial movement trajectories was highest, suggesting high planning noise. Our results suggest that workspace redundancy can modulate motor adaptation in a spatially specific manner, however, this modulation may depend on the level of planning noise.

**NEW & NOTEWORTHY** Interindividual differences in motor adaptation are partly explained by differences in movement variability. Movement variability is higher in a redundant workspace. Can workspace redundancy increase adaptation? In a within-subject experiment, we show that moving in a workspace that permits versus constrains movement variability in a given spatial dimension modulates adaptation rate in that dimension, at least in part of the workspace where initial movement trajectories vary most, indicating planning noise. Redundant workspaces might aid rehabilitation.

## Graphical abstract

**Figure:**
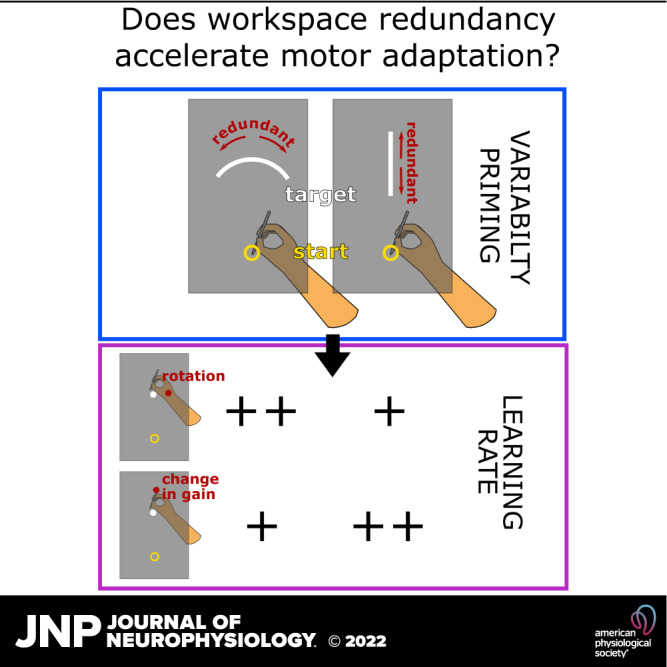

## INTRODUCTION

Movements vary across repetitions, even if intended to be identical and even after extensive training (1). Such variability can impede performance in tasks requiring high precision. Mechanisms by which the sensorimotor system minimizes detrimental effects of variability have therefore received considerable attention (2–4).

However, behavioral variability can also benefit future performance. Wu et al. (5) have shown that variability in motor output can accelerate human motor learning. They demonstrated that interindividual differences in the rate of reinforcement learning, as well as error-based learning, can be explained by differences in the amount of movement variability at baseline. Differences in baseline movement variability also explained differences in learning rates typically observed across tasks. Furthermore, Wu et al. (5) showed that movement variability can be reshaped by repeated exposure to a consistent dynamic perturbation, such that variability after exposure is enhanced along dimensions that were previously task-relevant.

A beneficial effect of movement variability on the rate of motor adaptation has also been shown by Singh et al. (6). In their study, interindividual differences in baseline variability in joint angles during reaching predicted differences in adaptation to altered kinematics and dynamics. They also found that variability in joint angles could explain differences in learning rates between dominant and nondominant hands.

Wu et al. (5) and Singh et al. (6) focused on differences in learning rate between individuals and between tasks. Singh et al. (6) speculated that “redundancy might be an idiosyncratic feature that is unique to each subject and/or session” (p. 14417). However, to help individuals optimize learning, e.g., during rehabilitation, effects of movement variability on learning would ideally be controllable within-subject. Wu et al. (5) demonstrated that movement variability can be enhanced for one and the same subject, through repeated exposure to a dynamic perturbation. However, they observed this reshaping of variability after learning and without any further exposure to the relevant learning task. It is, thus, yet unclear whether the aforementioned reshaping provides a way to improve learning within subjects.

Movement variability has been categorized either as noise arising from the periphery of the motor and musculoskeletal system during movement execution (7) or as a variability that emerges during motor planning (8, 9). The latter, but not the former, has been regarded as a substrate for motor learning (10). A simple experimental manipulation that can systematically alter movement variability due to motor planning in one and the same subject is a change in workspace redundancy, i.e., in the shape and spatial extent of targets (9, 11). Van Beers et al. (9) have demonstrated that endpoints of movements to visual targets with a redundant, less task-relevant spatial dimension (e.g., arc-shaped targets) display a random walk along this redundant dimension. Van Beers et al. (9) attributed this random walk to motor planning, based on a “planned aim point correction” model (4), in which planning noise accumulates across repetitions. Furthermore, it has been argued that movement variability along the less task-relevant dimension of redundant targets may represent active motor exploration (9, 11).

Here, we investigated whether movement variability shaped by workspace redundancy can influence motor adaptation in one and the same individual. To this end, we designed a within-subject experiment with an initial priming phase, in which two-dimensional targets for reaching had the shape of an arc, or a radially oriented line, allowing for variability in movement direction versus movement extent, respectively (9). In a subsequent test phase, we tested (single-trial) adaptation of movement direction and movement extent as a function of target shape during the preceding priming phase. Thus, our experiment examined whether the level of movement variability is more than an idiosyncratic feature of an individual determining that person’s ability or inability to learn. Instead, we hypothesized that movement variability can be harnessed to enhance learning in one and the same subject, through a simple intervention that exploits workspace redundancy.

## MATERIALS AND METHODS

### Subjects

We recruited 36 healthy volunteers via participant databases and among colleagues and students of Otto-von-Guericke University. Three volunteers were excluded from analyses due to a misunderstanding that resulted in strongly curved movements in one condition (one participant), or due to a high number of movements that were slower than an a priori defined velocity threshold, used for providing error feedback to participants during the experiment (two participants; >90% and >25% of trials in one condition). Of the remaining 33 participants, 10 were females. The age range was 21–32 yr (mean 25.3 yr, SD 2.4 yr). Thirty of the included participants were right-handed, as confirmed via the Edinburgh handedness inventory (EHI; 12). Three participants fulfilled criteria for ambidexterity (EHI between −40 and +40). All participants gave written informed consent. The study was approved by the ethics committee of Otto-von-Guericke University and conducted in accordance with the Declaration of Helsinki. All volunteers received reimbursement for participation (8 Euros/h).

### Apparatus

**Figure 1.**
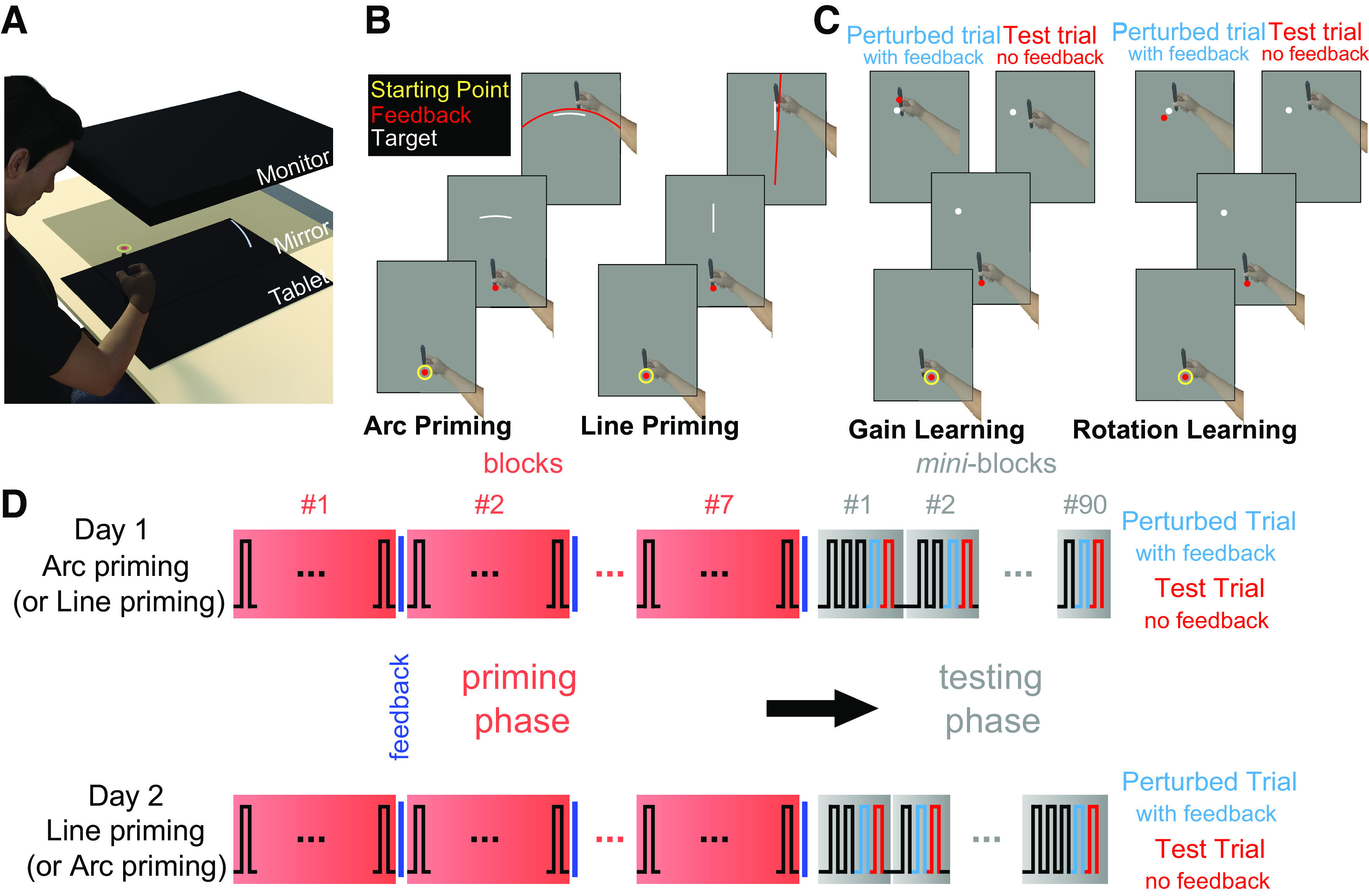
Experimental setup and task. *A*: experimental table with three levels. For illustration purposes, the middle level is shown transparent, while it was actually opaque, hiding the hand on the lower level. The apron, headphones, and polarizing sunglasses participants were wearing are not shown. *B*: schematic of a single trial of the priming phase, for arc-priming (*left*) and line-priming (*right*). Arc-priming and line-priming were completed by each participant, but in separate sessions a week apart. Their order was counterbalanced across participants. The endpoint feedback displayed here was only shown in 25% of trials during the priming phase. *C*: schematic of the penultimate trial (“perturbed trial”) and last trial (“test trial”) of a mini-block during the test phase, shown separately for gain learning (*left*) and rotation learning (*right*). Only the last two trials of two exemplary mini-blocks are displayed. *D*: schematic of each experimental session (*day 1* and *day 2*), separated by 1 wk. Each participant first completed seven blocks of priming (60 trials each), which differed between experimental sessions, before completing an adaptation test phase, which was identical for the two sessions. The test phase was divided into 90 mini-blocks, each consisting of three, four, or five consecutive reaches to the same target. Visual endpoint feedback was provided only during the penultimate (“perturbed”) trial. Single-trial adaptation was tested on the last (“test”) trial of each mini-block.

Participants sat in a dimly lit room at an experimental table that had three levels (Fig. 1*A*). At the lowermost level, there was a graphics tablet (Intuos 4XL, Wacom, Japan; 5,080 lines per inch, sampled at 200 Hz, active area of 48.8 × 30.5 cm). During the experiment, participants moved a stylus across the graphics tablet with their right hand. They could not see their hand because it was hidden underneath the middle level of the experimental table. In addition, arms and shoulders were hidden from sight underneath a black apron, which participants wore around the neck, and whose other end was attached to the middle level of the table. An LCD monitor at the uppermost level, facing downward, provided visual feedback of a home position, targets, and movement endpoints. Participants could see this feedback in a mirror placed at the middle level of the experimental table, facing upward. The distances between the monitor and the mirror, and between the mirror and the tablet, were identical, creating the illusion that the home position, targets, and endpoint feedback appeared in the same plane as the tip of the stylus. Participants wore polarizing sunglasses, which minimized visibility of a second, slightly offset reflection of the monitor at the glass surface of the mirror, as well as headphones (for auditory feedback on movement speed, see *Priming phase in Experimental Task section*).

### Experimental Task

The experimental task was programmed and presented using Presentation (Neurobehavioral Systems). Participants had to move the stylus rapidly from a home position to a target and stop as accurately as possible on the target. Each participant completed two sessions, separated by 1 wk. Each session included an initial priming phase, followed by a test phase. The test phase was identical across sessions, while the priming phase differed between sessions.

#### Priming phase.

The priming phase was designed to allow for movement variability along one spatial dimension of movements, i.e., movement extent or movement direction, while constraining movement variability along the other dimension, respectively. This was achieved by controlling the shape of targets during the priming phase. In a given experimental session, targets during priming were either an arc, centered on the home position (arc-priming; Fig. 1*B**, left*), or a straight line, radially oriented relative to the home position (line-priming; Fig. 1*B**, right*). For both arc- and line-priming, participants were instructed to stop as accurately as possible on the target. Arcs and lines both had a length of 8 cm (=arc length in case of arcs). Arc-priming thus permitted variability in movement direction, whereas line-priming permitted variability in movement extent. Written instructions emphasized that it was not important where on the arc, and where on the line, participants would stop their movement, as long as they stopped on the target. As a result, arc-priming emphasized precision of movement extent over movement direction, and line-priming emphasized precision of movement direction over movement extent. In both cases, targets (line width of 0.5 cm) were 15 cm away from the home position (in case of radial lines, 15 cm corresponded to the distance between the home position and the mid-point of the line). In any given trial, a target could be located straight ahead, or 30° clockwise, or counterclockwise, relative to straight ahead (in case of arc targets, 30° relative to straight ahead corresponded to the mid-point of the arc). We included three different target locations to emphasize motor planning noise, given that moving to a single, repeated target emphasizes motor execution noise (7). It has been proposed that planning noise, but not execution noise, may benefit learning (10). The home position was represented by a yellow ring (1 cm in diameter, line width of 0.4 cm), located in the midline of the tablet, roughly aligned with the body midline.

Each trial started with the presentation of the home position, prompting participants to move the stylus toward the home position. When the stylus was only 2 cm away from the home position, a red cursor (0.8 cm in diameter) appeared, whose location tracked the location of the stylus. Participants moved the red dot into the home position. After 800 to 1,200 ms (uniform random distribution), a target was presented at one of the three abovementioned locations. During arc-priming, the target was always an arc. During line priming, the target was always a straight line. Participants were instructed to produce a single, rapid reaching movement to the target, and to stop, as accurately as possible, on the target (regardless of where exactly they would stop on the target, see above). There was no time constraint for movement initiation. Once the stylus left the home position, the red cursor and the home position were extinguished. Movement offset was defined as the first time since movement initiation when no further change in x- and y-coordinates of the stylus was detectable for at least 33.3 ms (two vertical refresh rates of the monitor). If average movement speed (from leaving the home position to movement offset) was <20 cm/s, an error sound was presented via the headphones. One second after movement offset, the home position reappeared, prompting participants to return to the home position.

Two types of feedback emphasized movement accuracy along the highly task-relevant spatial dimension defined by arc-shaped targets (highly task-relevant: movement extent) or line-shaped targets (highly task-relevant: movement direction). In 25% of priming trials (pseudorandomly selected), participants received feedback on their movement endpoint, but only for one spatial dimension (13). During arc-priming, participants saw a red circle centered on the home position, whose radius corresponded to the radial distance of their movement endpoint from the home position (Fig. 1*B**, left*). During line-priming, participants saw a red line, originating from the home position and extending beyond the screen limits, whose angular orientation corresponded to the angular direction of the movement endpoint relative to the home position (Fig. 1*B**, right*). As a result, participants received visual feedback for movement extent, but not movement direction, during arc-priming, and vice versa during line-priming. Participants were instructed to make the red circle overlie the arc-shaped target during arc-priming, and the red radial line overlie the line-shaped target during line-priming. This endpoint feedback thus emphasized the precision of movement extent over direction for arc-priming and vice versa for line-priming. Endpoint feedback was presented for a duration of 1 s following movement offset. During piloting, we observed that such one-dimensional endpoint feedback enhanced priming effects on movement variability. However, we were concerned that we might emphasize the task relevance of one dimension too strongly, and thereby enhance learning from perturbed feedback in that dimension during the later test phase, e.g., by strongly drawing attention to that dimension. This might create a pattern of learning during the test phase opposite to the effect we expected movement variability in the task-irrelevant dimension to have on learning. We, therefore, opted to provide endpoint feedback only sometimes, i.e., in 25% of priming trials, instead of in each individual trial.

In addition, after each block of the priming phase (60 trials each), participants received feedback on how close to the targets they had stopped, on average, across the preceding block. Specifically, during arc-priming, a message on the screen informed them by how many centimeters they had over- or undershot the arc-shaped targets, whereas no feedback on movement direction was provided. During line-priming, the message informed them by how many degrees they had missed the line-shaped targets, whereas no feedback on movement extent was provided. In both cases, feedback was unsigned (i.e., the mean of absolute values across trials).

#### Test phase.

While the priming phase was designed to permit movement variability in one spatial dimension but not the other, the test phase examined whether these spatially specific changes to movement variability would influence motor adaptation in a spatially specific manner. To this end, the test phase repeatedly probed how strongly participants adapted following a single trial with a visuomotor perturbation (14), either a rotation or a change in gain. We chose to examine adaptation following a single perturbed trial, rather than during extended periods of a constant perturbation because this allowed us to interleave trials testing for adaptation in both spatial dimensions. This was important as we did not expect priming to have long-lasting effects. Following the idea that movement variability can enhance motor adaptation (5, 6), we expected stronger adaptation to visuomotor rotations following arc-priming, compared with line-priming, and vice versa for adaptation to changes in gain. To examine this, the test phase differed from the priming phase in three ways, as follows.

First, targets were no longer arc-shaped or line-shaped, but consisted of a white dot (0.8 cm in diameter), placed 15 cm away from the home position, either straight ahead, or 30° clockwise or counterclockwise of straight ahead. This dot target ensured that both spatial dimensions were equally task-relevant.

Second, the order in which targets appeared at specific locations (straight ahead, or 30° rotated clockwise or counterclockwise) was structured across the test session. Specifically, there were mini-blocks of three, four, or five consecutive trials for which target location remained constant (within each mini-block).

Third, endpoint feedback was provided only in the penultimate trial of each mini-block (henceforth called “perturbed trial”, see Fig. 1*C*) and differed from the priming phase. Following movement offset in the penultimate trial of each mini-block, a red, static cursor was presented, either at the location of the true movement endpoint, or in a location that was defined by a rotational or gain perturbation, applied to the true endpoint location. Specifically, in some trials, the cursor was either rotated away from the true end point by 8.53° or by 5.71°, each either clockwise or counterclockwise, keeping radial distance from the home position veridical. In other trials, the radial distance between the true movement endpoint and the home position was multiplied by 1.15, 1.1, 0.9, or 0.85 to compute the radial distance at which the cursor was shown, relative to the home position, keeping angular direction veridical. We chose these specific values for rotations and gain perturbations because they would produce similar distances between the cursor and the true movement endpoint for rotation versus gain, assuming a movement extent of 15 cm (as required by the target). For a movement extent of 15 cm, a rotation by 8.53° or by 5.71° would move the cursor by 2.25 cm or 1.5 cm, respectively, in a direction that is orthogonal to the direction of movement. Similarly, a gain of 1.15, or 0.85, would move the cursor by 2.25 cm along the direction of movement, whereas a gain of 1.1, or of 0.9, would move the cursor by 1.5 cm.

### Experimental Procedure

Each participant underwent arc-priming in one experimental session and line-priming in the other session (Fig. 1*D*). The order of arc- versus line-priming was counterbalanced across subjects. In each experimental session, the priming phase consisted of seven blocks of 60 trials each. Across the priming phase, targets were presented equally often at each of the three possible locations (straight ahead, 30° clockwise, or counterclockwise, relative to straight ahead), in a pseudorandomized order.

The test phase was identical between experimental sessions. Each test phase consisted of 90 mini-blocks. Each of the eight possible perturbations (rotation by ±8.53°, or by ±5.71°, and gain change by ±0.15, or by ±0.1) was presented in nine mini-blocks. A rotation was never combined with a gain perturbation. In an additional 18 mini-blocks, endpoint feedback in the penultimate trial was veridical. Mini-blocks of three, four, or five trials were equally frequent, and equally often associated with each perturbation, and, independently, with each of the three target locations. The order of mini-blocks was pseudorandomized anew for each participant but kept constant across the two sessions for each participant. Each of the eight possible perturbations had to be presented once, and two additional mini-blocks with nonperturbed trials had to be presented before a perturbation was repeated. There was a break after 30 and 60 mini-blocks. Block-wise feedback on movement accuracy, as provided during priming, was not present during the test phase.

The entire test phase took, on average, 26 min and 14 s, and included 360 movements.

### Data Analysis and Statistics

Data were analyzed in MATLAB (Mathworks) using custom-written scripts. Statistical analyses were run in JASP (15) and MATLAB. We excluded trials from the priming and test phases based on three criteria. First, we excluded trials in which an error sound was presented because movements were too slow [0.97 ± 1.93% of trials (range 0–10%) during arc-priming, 1.62 ± 1.76% of trials (range 0–5.7%) during line-priming, and 1.34 ± 1.74% of trials (range 0–7.8%) during test (across both sessions; means ± SD)]. Second, we excluded trials if movements were strongly curved. To this end, trials were excluded in which the maximum normal distance to a straight-line connection between home position and movement endpoint exceeded a value of 2 cm [0.1 ± 0.42% of trials (range 0–2.38%) during arc-priming, 0.05 ± 0.18% of trials (range 0–0.71%) during line-priming, and 0.05 ± 0.09% of trials (range 0–0.42%) during test (across both sessions; means ± SD)]. Third, we excluded trials in which movement endpoints were far away from the distribution of all movement endpoints, computed per subject, target location, and condition. Specifically, movements whose endpoints were more than eight times the interquartile range away from the median endpoint (Euclidean distance) were excluded [0.15 ± 0.27% of trials (range 0–1.19%) during arc-priming, 0.04 ± 0.09% of trials (range 0–0.24%) during line-priming, and 0.06 ± 0.14% of trials (range 0–0.56%) during test (across both sessions; means ± SD)]. All outliers defined in this way, as well as all trials excluded based on movement curvature, were further confirmed via visual inspection (see Supplemental Figs. S1 and S2; all Supplemental material is available at https://doi.org/10.5281/zenodo.6980246). Importantly, all statistical results are qualitatively unchanged when no trials were excluded based on movement curvature. We did not recompute statistical results when no trials were excluded as outliers because visual inspection revealed that outliers were clearly gross movement errors (including movements to a wrong target location, or movements whose endpoints remained in the vicinity of the home position, see Supplemental Figs. S1 and S2).

Given that subjects’ task was to stop on the target as accurately as possible, we computed movement direction as the angle between movement endpoint and home position. For a separate analysis, which tested for variability in initial movement direction, we recomputed movement direction at the time of peak movement velocity, computed from the derivative of stylus position after smoothing with a second-order Savitzky-Golay filter [frame length of 31 samples, corresponding to 155 ms (16)]. Movement extent was computed as the Euclidean distance between movement endpoints and the home position. To quantify variability in movement direction (in degrees) and movement extent (in cm), we computed interquartile range (IQR) as a robust measure of spread. To avoid any influence of target-specific spatial biases in reaching, IQR was computed separately for each target location and then averaged across target locations. To achieve identical units for variability in direction versus extent, and compare them in the same statistical model, IQR values were reexpressed as a percentage. Specifically, IQR for movement direction was divided by 30.56° (i.e., the central angle of arc-shaped targets), and then multiplied by 100. IQR values for movement extent were divided by 8 cm (i.e., the length of radial lines) and then multiplied by 100. Resulting values thus represent variability in percent, where 100% would mean that a participant fully exploited the possibility to vary endpoints along the full length of arc- and line-shaped targets. We call this percentage “explored variability” throughout the manuscript. Explored variability was statistically compared between sessions (arc-priming vs. line-priming) and dimensions (movement direction and extent) in a 2×2 repeated-measures ANOVA. For the test phase, explored variability was computed analogously, based on all trials except the last trial in each mini-block (because movements in the last trial were expected to reflect movement corrections in response to visual feedback in the preceding trial). Planned follow-up comparisons via dependent samples, two-tailed *t* tests were Bonferroni-corrected.

We expected that participants would adapt movements in the last trial of each mini-block (henceforth called “test trial,” see Fig. 1*C*) to the direction and magnitude of the error introduced by the visuomotor perturbation in the preceding trial (“perturbed trial” in Fig. 1*C*).

We, therefore, regressed the change in movement endpoint from perturbed trials to test trials on the (signed) perturbation in the perturbed trial. The rate of adaptation is reflected in the slope of the regression line (14).

We predicted stronger adaptation of movement direction from perturbed trials to test trials after arc-priming, compared with line-priming, and vice versa for adaptation of movement extent. Our hypothesis was thus a three-way interaction effect on movement corrections between the perturbation size (signed) in the perturbed trial, the preceding priming condition (arc- vs. line-priming), and spatial dimension (movement direction versus extent). To test this interaction, we ran a multiple linear regression model for each individual, and then tested regression coefficients at the group level (two-sided, one-sample *t* test against zero). To compare spatial dimensions (movement direction vs. extent) in the same model, we standardized (z-transformed) our response variable (changes in movement direction and extent from the perturbed to the test trial). The multilinear regression model thus included, as predictors, the signed perturbation size (in cm, see above), priming (dummy-coded, arc- vs. line-priming), and spatial dimension (movement direction vs. extent, dummy-coded), as well as all two-way interactions, and, importantly, the three-way interaction. Simple effects of priming, dimension, and all two-way interactions were included to test the three-way interaction, but were not our primary interest. To avoid any interference between a perturbation in one spatial dimension, and movement adjustments in the other, changes to movement direction from a perturbed trial to a test trial were ignored when a gain perturbation was applied in a perturbed trial, and changes to movement extent were ignored when a rotation was applied.

Target location was not an a priori factor of interest. However, observing a trend-level three-way interaction when collapsing across all three target locations, we repeated the above analysis for each of the three target locations separately, Bonferroni-correcting resulting *P* values. The rationale for target-wise regression analysis testing for priming effects on learning was that the structure of movement variability, including the amount of initial variability suggestive of planning noise, is known to vary across the workspace (7).

In the case of significant three-way interactions, we conducted planned comparisons in two linear regression models, each testing for one spatial dimension separately. These models included, as predictors, the signed perturbation size (in cm), priming, and their interaction. Given that movement extent and direction were tested in separate models, we did not standardize the response variable (statistical results are qualitatively unchanged with standardization). Regression coefficients extracted from each subject for the interaction term were then tested against zero at the group-level via two-tailed one-sample *t* tests, and *P* values were Bonferroni-corrected.

## RESULTS

### Movement Variability

**Figure 2.**
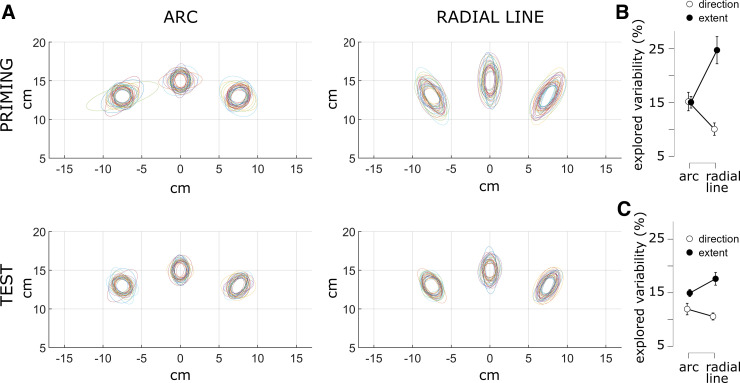
Movement variability during priming and test phases. *A*: 68% confidence ellipses (∼1 standard deviation) for each participant (coded by color), centered on the three target locations, both for the priming phase (*upper*) and the test phase (*lower*). The left column shows variability related to arc-priming. The right column shows variability related to line-priming. Explored variability (in %) as a function of spatial dimension (movement direction vs. extent) and priming, for the priming phase (*B*) and test phase (*C*). Error bars represent 95% confidence intervals. *n* = 33 subjects, 10 female.

We expected higher variability of movement direction during arc-priming, compared with line-priming, and higher variability of movement extent during line-priming, compared with arc-priming. Supplemental Figs. S1 and S2 show movement endpoints across all trials for each participant, for the priming and test phase, respectively. Mean endpoints for individual subjects varied across individuals and target locations. We, therefore, computed variability (IQR) for each individual and each target location separately, and then averaged across target locations. Figure 2*A* displays individual subjects’ endpoint variability as 68% confidence ellipses (∼1 standard deviation) aligned with true target locations, for the priming phase (upper row) and test phase (lower row). For statistical analysis, individual subjects’ IQR values for movement extent and movement direction were reexpressed as explored variability, i.e., a percentage of maximum permitted variability along the redundant dimensions of arc- and line-shaped targets. Explored variability was then entered into a 2×2 repeated-measures ANOVA to test for the expected interaction between priming (arc- vs. line-priming) and spatial dimension (movement direction vs. extent).

We found a significant interaction effect on explored variability during the priming phase between priming and spatial dimension [*F*(1,32) = 101.3, *P* < 0.001, partial-η^2^ = 0.76; Fig. 2*B*]. Planned comparisons revealed that explored variability in movement direction was significantly higher during arc-priming, compared with line-priming [*t*(32) = 6.3, *P* < 0.001, d = 1.1, Bonferroni-corrected; means ± SD: 15.2 ± 4.9% vs. 10.1 ± 1.5%, corresponding to an IQR of 4.65 ± 1.5° vs. 3.08 ± 0.46°, for arc-priming vs. line-priming, respectively]. Conversely, explored variability in movement extent was significantly higher during line-priming, compared with arc-priming [*t*(32) = 7.1, *P* < 0.001, d = 1.2, Bonferroni-corrected; means ± SD: 24.7 ± 8.5% vs. 15 ± 3.6%, corresponding to an IQR of 1.98 ± 0.68 cm vs. 1.2 ± 0.29 cm, for line-priming vs. arc-priming, respectively].

Interestingly, we found a similar, significant interaction effect on explored variability during the test phase between priming and spatial dimension [*F*(1,32) = 51.7, *P* < 0.001, partial-η^2^ = 0.618; Fig. 2*C*]. Planned comparisons revealed that explored variability in movement direction was significantly higher during test following arc-priming, compared with line-priming [*t*(32) = 3.09, *P* = 0.008, d = 0.54, Bonferroni-corrected; means ± SD: 12 ± 2.8% vs. 10.6 ± 1.8%, corresponding to an IQR of 3.67 ± 0.86° vs. 3.24 ± 0.55°, for arc-priming vs. line-priming, respectively]. Conversely, explored variability in movement extent was significantly higher during test following line-priming, compared with arc-priming [*t*(32) = 4.88, *P* < 0.001, d = 0.85, Bonferroni-corrected; means ± SD: 17.6 ± 4.5% vs. 14.9 ± 3.2%, corresponding to an IQR of 1.4 ± 0.36 cm vs. 1.19 ± 0.26 cm, for line-priming vs. arc-priming, respectively].

### Motor Adaptation

We predicted stronger adaptation of movement direction following arc-priming, compared with line-priming, and vice versa for adaptation of movement extent. This would be evident in a three-way interaction between the (signed) perturbation size (in cm), priming, and spatial dimension.

When collapsing across all three target locations, we found a trend for a three-way interaction [*t*(32) = 1.7, *P* = 0.099, d = 0.3] in the expected direction (higher rate of learning from rotations after arc-priming, compared with line-priming). However, there was considerable variability with respect to regression model fit. *R*^2^ ranged from 0.02 to 0.41 (mean 0.16, SD 0.09). Considering only participants for whom the regression model explained motor corrections by more than 10% (*R*^2^ ≥ 0.1; 22 participants), the three-way interaction was significant [*t*(21) = 2.6, *P* = 0.02, d = 0.56]. Similar statistical results were obtained when choosing other thresholds for *R*^2^ [*t*(30) = 2, *P* = 0.05, d = 0.36, for *R*^2^ ≥ 0.05; *t*(15) = 3.1, *P* = 0.007, d = 0.78, for *R*^2^ ≥ 0.15; *t*(7) = 4.6, *P* = 0.003, d = 1.6, for *R*^2^ ≥ 0.2]. Subjects for whom the signed perturbation size, priming, spatial dimension, and their two- and three-way interactions explained motor corrections to a substantial degree, hence showed the expected priming effect on learning.

**Figure 3.**
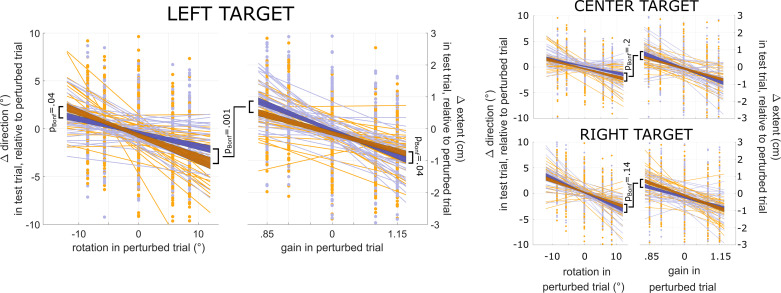
Single-trial motor adaptation (from the perturbed trial to the test trial). The figure shows learning at each of the three target locations. For each target location, the *left* shows rotation learning, and the *right* shows gain learning. In each panel, the *x*-axis represents signed perturbation size, and the *y*-axis represents change in behavior from the perturbed to the test trial [movement direction for rotation learning (left *y*-axis), and movement extent for gain learning (right *y*-axis)]. In all panels, yellow and purple dots represent individual-trial data points across subjects. Thin yellow and purple lines represent individual-subject regression lines for arc-priming and line-priming, respectively. Dark yellow and dark purple shades represent mean ± standard error of the mean of regression lines across all subjects (yellow = arc-priming, purple = line-priming). For statistical analyses, regressions were computed on a single-subject level, and regression coefficients were then subjected to group-level statistics (two-sided, one-sample *t* test against zero; *P* values were Bonferroni-corrected). Positive rotation values on the *x*-axis (*left*) correspond to clockwise rotations. Positive differences in direction (left *y*-axis) indicate that movement direction was more clockwise in the test trial compared with the perturbed trial. Negative changes to movement extent (right *y*-axis) correspond to shorter movements in the test trial, relative to the perturbed trial. *n* = 33 subjects, 10 female.

**Figure 4.**
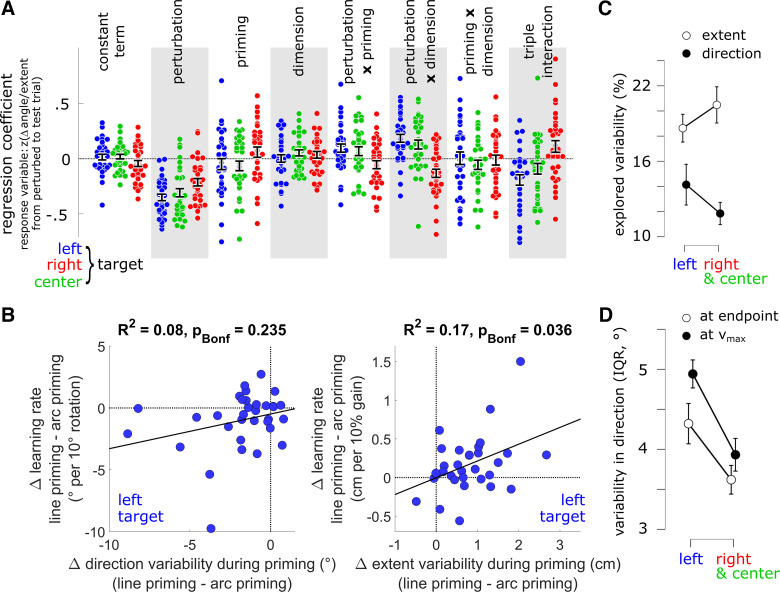
Regression coefficients and movement variability by target location and interindividual correlation between variability and learning. *A*: regression coefficients for individual subjects (dots) and target locations (colors) for each of the six predictors and the constant term of the regression model. Error bars represent standard error of the mean, centered on the mean across subjects. *B*: interindividual correlation between priming effects on learning (*y*-axis) and priming effects on variability during the priming phase (*x*-axis), both at the left target. *Left*: for movement direction (computed at movement endpoint); *right*: for movement extent. *P* values, obtained from Pearson’s correlation, are Bonferroni-corrected. *C*: explored variability in movement direction vs. extent at the left vs. the other two target locations during the priming phase. *D*: variability in movement direction, computed either at the endpoint, or at the time of maximum velocity (v_max_), shown separately for the left target location and the other two target locations (right and center). Error bars in *C* and *D* represent 95% confidence intervals. *n* = 33 subjects, 10 female.

We explored another potential explanation for why priming effects on learning did not reach statistical significance when computed across the entire group of participants. Movement variability is not uniform across the workspace (7). Given the proposed link to learning (10), it is possible that the effects of priming variability on learning, too, varied across the workspace. We, therefore, examined the effect of priming on learning at each of the three target locations separately (correcting for multiple comparisons). We found a significant three-way interaction for the left target [*t*(32) = 4, *P* = 0.001, d = 0.7, Bonferroni-corrected; Fig. 3*, left*], but not for the other two target locations [*t*(32) = 1.9, *P* = 0.22, d = 0.32, for the center target, and *t*(32) = 2.1, *P* = 0.14, d = 0.36, for the right target; Bonferroni-corrected; Fig. 3*, right*; see also Fig. 4*A*, column titled “triple interaction”].

For the left target, follow-up regression models for each spatial dimension separately revealed significantly higher rotation learning rates after arc-priming, compared with line-priming [*t*(32) = 2.5, *P* = 0.04, d = 0.54, Bonferroni-corrected], and significantly higher gain learning rates after line-priming, compared with arc-priming [*t*(32) = 2.5, *P* = 0.04, d = 0.58, Bonferroni-corrected]. Models had a mean *R*^2^ of 0.22 (SD: 0.056).

Interestingly, we observed a significant positive correlation between priming effects on gain learning versus priming effects on movement extent variability (*R*^2^ = 0.17, *P* = 0.036, Bonferroni-corrected; Fig. 4*B**, right*). The more variable movement extent became during line-priming, the higher the subsequent increase in gain learning rate, compared with arc-priming, both computed at the left target location. The correlation between priming effects on rotation learning versus priming effects on movement direction variability was positive, but not significant (Fig. 4*B**, left*), at least when variability in direction was computed at movement endpoints (see below).

Why was a significant priming effect on learning observed at the left target location? Van Beers (7) observed that variability of initial movement direction (i.e., within the first centimeter of movement) was larger for target locations in the upper left quadrant than in the upper right quadrant (Fig. 4*D* in Ref. 7). In adaptation studies, initial movement direction serves as a more direct readout of feedforward planning than endpoint direction (e.g., Refs. 17–19). Higher initial movement variability in one part of the workspace would thus be consistent with higher variability in planning. Given Dhawale et al.’s (10) proposal that it is variability in planning that benefits learning, we compared movement variability, both at movement endpoints and at the time of maximum velocity, between the left target location, for which we observed a significant priming effect on learning, and the other two target locations.

We found that variability in direction at movement endpoint was larger for the left target location compared with the other two target locations, whereas variability in movement extent was lower [Fig. 4*C*; *F*(1,32) = 33.1, *P* = 0.001, partial-η^2^ = 0.51, for the dimension × target location interaction; *t*(32) = 3.5, *P* = 0.002, d = 0.61, for the difference in extent variability between target locations; *t*(32) = 4.1, *P* < 0.001, d = 0.71, for the difference in direction variability between target locations; Bonferroni-corrected; compare with Fig. 4*A* in Ref. 7]. Importantly, this difference in direction variability between target locations was particularly pronounced when movement direction was computed at the time of maximum velocity [Fig. 4*D*; *F*(1,32) = 8.8, *P* = 0.006, partial-η^2^ = 0.22, for the interaction between target location and movement stage (initial movement vs. endpoint)]. The effect of priming on learning rate (Fig. 3) was thus observed at the target location that showed the largest initial movement variability. Indeed, the correlation between priming effects on rotation learning versus priming effects on movement direction variability (Fig. 4*B*, *left*) was significant when direction variability was computed at the time of maximum velocity (*R*^2^ = 0.14, *P* = 0.03). The more variable initial movement direction became during arc-priming, compared with line-priming, the higher the subsequent increase in rotation learning rate.

## DISCUSSION

We provide first evidence that repeated reaching to targets that have a redundant spatial dimension can enhance (single-trial) visuomotor adaptation along that dimension, relative to adaptation after reaching to targets with little redundancy, at least in part of the workspace. We attribute this learning benefit to a systematic change in movement variability, which was higher in each target dimension when that dimension was redundant versus nonredundant, both during priming (9), and, to a lesser degree, during the subsequent test phase. Although previous studies have shown that interindividual differences in motor adaptation correlate with differences in movement variability (5, 6), our study is the first to provide proof of principle that learning can be modulated for a given individual, and for the same effector and task, by introducing redundancy in the target that allows for movement variability, at least for part of the workspace. Our study also shows that this effect on learning is spatially specific. A simple way to enhance learning intraindividually might thus be through workspace redundancy, e.g., for rehabilitation. We discuss potential mechanisms by which workspace redundancy, and/or consequent movement variability, may enhance motor adaptation, alongside limitations and open questions of our study, as well as potential future directions.

Although variability in behavior poses a challenge to the sensorimotor system (2–4), it can also benefit performance. Reinforcement learning, for example, involves exploration (18), i.e., variation of behavior. Reinforcement learning has inspired research in songbirds providing evidence that behavioral variability can promote motor learning. When specific variants in their song are negatively reinforced, zebra finches adjust their song accordingly. This suggests that such variants do not purely stem from inevitable, uncontrollable noise, but instead reflect motor exploration (20). Indeed, variability in song during learning is actively produced in a basal-ganglia-related circuit, whose silencing results in highly stereotyped songs (19). Such variability is actively regulated depending on the requirements of the task at hand (21). Wu et al. (5) and Singh et al. (6) have shown that variability in motor output can benefit motor learning also in humans and beyond reinforcement learning.

Variability in motor output has been attributed to three compartments and sources in each compartment at the level of molecules, cells, synapses, and networks (22). Movement variability can result from noise arising in the periphery of the motor and musculoskeletal system, called execution noise (7, 23). In addition, noise and variability during planning (4, 8, 9), and noise “upstream” of the motor system, within sensory systems (22), also give rise to movement variability.

Comparing conditions with different levels of execution noise, He et al. (14) found no consistent change in (single-trial) motor adaptation rate. For example, they compared adaptation of right-hand movements to targets at different distances. Movement variability increases with target distance (14), an effect that has been attributed to execution noise (7). Furthermore, He et al. (14) provided visual feedback on movement endpoints throughout the baseline session. Visual feedback allows for trial-to-trial movement corrections that may converge over time, and thereby eventually reduce variability in movement planning. Because of this, Dhawale et al. (10) argued that within-subject differences in baseline movement variability observed by He et al. (14) may have largely reflected execution noise.

Learning, however, may benefit from variability that arises from planning noise. As “planning noise” arises within the central nervous system, signals corrupted by planning noise are, in principle, directly available within the central nervous system, and can be “stored” for adjusting plans for future motor output (4, 9). Execution noise, on the other hand, is detectable by an agent only via her sensory systems, which are themselves corrupted by noise. Accordingly, it has been proposed that learning from movement error differentiates between (estimated) proportions of error that arise from planning noise versus execution noise and corrects for a larger fraction of an error in case of a larger contribution of planning versus execution noise (4). A similar conclusion that planning noise should increase learning from error, while execution noise should decrease learning, follows from Kalman filter theory (24), as shown in simulations and based on modeling of kinematics in the study by van der Vliet et al. (25). Indeed, increasing uncertainty about the current visuomotor mapping, and thereby variability during planning, enhances motor adaptation (26). Taken together, planning noise, but not execution noise, is thought to benefit learning (10).

Our focus on workspace redundancy was motivated by this idea. Workspace redundancy is considered to permit movement variability that arises at a planning stage (9, 11). Van Beers et al. (9) have shown that movements to targets with a redundant spatial dimension display a random walk of endpoints along this dimension, i.e., a positive lag-1 autocorrelation. They explained this serial dependence across consecutive movements by iterative shifts to an aim point, as assumed in van Beers’ “planned aim point correction model” (4). This iterative shift is possible because motor commands corrupted by planning noise, but not execution noise, are directly available to the central nervous system, specifically to compute subsequent aim points. Accordingly, the random walk along redundant target dimensions observed by van Beers et al. (9) speaks in favor of a central origin of this variability. In line with the idea that workspace redundancy permits movement variability arising from planning, Cashaback et al. (11) have shown that humans adjust reaching movements to task-irrelevant dynamic perturbations pushing movement endpoints into less explored sections of a redundant target, without relevance to goal achievement. Specifically, Cashaback et al. (11) observed movement adjustments such that movements ended further downstream of the perturbation, effectively covering a different portion of the target than without perturbation. These adjustments were already observed in the movement path before a perturbation took effect, thus reflecting adjusted planning.

We exploited the opportunity afforded by workspace redundancy to permit movement variability due to planning noise in a spatially specific way, with the aim to test whether this modulates motor adaptation. To further avoid stereotyped planning and, thus, an emphasis on execution noise (7), we varied target locations across trials. Van Beers et al. (9) and Cashaback et al. (11) both proposed that variability in planning due to workspace redundancy may (partly) reflect exploration. Exploration afforded by workspace redundancy could explain priming effects on subsequent learning in our study. Having explored varying directions during arc-priming, for example, may have facilitated exploratory changes to movement direction aimed at compensating for directional errors during subsequent test. Participants may have “felt more comfortable” to change to a movement direction they had “tried out” before. Our finding of a prominent priming effect on learning at the left target location, where initial movement variability was largest, is consistent with the idea that it is variability in planning, not execution, which benefits learning (10). Following previous observations in perceptual learning (27) and motor adaptation (28), our study provides further evidence that task-irrelevant aspects of a task can nevertheless influence later performance.

Conversely, precise movement directions during line-priming may have hampered later exploration of movement direction, reflecting a “lack of previous experience” with movement directions other than those required by line-shaped targets. Use-dependent learning could also explain the observed pattern of learning rates. Use-dependent learning biases movements to become more similar to previous movements (13, 29). Use-dependent learning may have strongly biased movement directions toward the orientations of line-shaped targets, and movement extent toward the distance of arc-shaped targets. If this bias persisted during the test phase, it may have limited adaptability of movements during test. Indeed, use-dependent learning can act to counter the effects of error-based learning (13).

So far, we have assumed that workspace redundancy influences learning via movement variability. However, it is possible that movement variability and learning are both influenced by workspace redundancy, but independently of each other. By definition, workspace redundancy determines task relevance of spatial dimensions. Task relevance guides attention. It is possible that participants paid more attention to error along spatial dimensions that had been highly task-relevant during priming. However, if this tuning of attention to task-relevant spatial dimensions influenced subsequent learning, a pattern of priming effects on learning would be expected that is opposite to what we observed (more learning along the spatial dimension that had previously been highly task-relevant, and, therefore, attended). On the other hand, it is possible that the less precise spatial dimension of movements during test received more attention. Given that spatially specific priming effects on variability persisted into the subsequent test phase, this would predict more attention to movement direction following arc-priming and more attention to movement extent following line-priming. Such a shift in attention could, in principle, explain the observed priming effects on learning, for example, if cognitive strategies, such as strategic reaiming (30), played a major role in the observed learning. Endpoint feedback, as provided here, typically promotes explicit over implicit learning (31). Cognitive strategies typically increase response times and are disrupted when movement initiation is speeded (32). Comparing response times, computed from the time of target presentation to movement initiation, between the perturbed and test trial revealed that response times decreased significantly from the perturbed to the test trial [*t*(32) = 4.4, *P* < 0.001, paired-samples *t* test; from 483.8 to 460.8 ms]. There was no evidence that the difference in response time from the perturbed to the test trial correlated with the amount of motor correction, neither in movement direction [mean *R*^2^ across subjects 0.032, *t*(32) = 0.5, *P* = 0.6] nor in movement extent [mean *R*^2^ across subjects 0.033, *t*(32) = 0.3, *P* = 0.8; one-sample *t* tests against zero of Fisher z-transformed correlation coefficients, each computed within subjects]. The role of attention, and of explicit versus implicit learning, for the effects of workspace redundancy on learning observed here could be subject to future investigation.

As pointed out by one of our reviewers, our manipulation of target shape during the priming phase may have influenced target perception during the subsequent test phase, such that the dot that served as a target in the test phase was perceived (slightly) arc-shaped following arc-priming and (slightly) vertically elongated following line-priming. However, if this played a major role, one might expect a pattern of learning that is opposite to what we observed. For example, a bias to perceive the dot target as arc-shaped would have reduced the angular target error for rotated feedback (discrepancy between perceived target and rotated endpoint feedback). Given that target error can drive learning, one might thus expect (slightly) reduced learning from rotation following arc-priming and (slightly) reduced learning from a change in gain following line-priming. This, however, is the opposite of what we observe, we find stronger rotation learning following arc-priming, compared with line-priming, and stronger gain learning following line-priming, compared with arc-priming. Hence, if we had asked participants to bisect targets during priming, abolishing differences in movement variability while retaining any perceptual carry-over effects on target shape during the test phase, we would expect either no priming effect on learning or the opposite pattern from what we observe.

One limitation of our study is that the predicted three-way interaction effect between perturbation, priming, and spatial dimension, was only a statistical trend when computed across all target locations and all subjects. However, inspection of the coefficient of determination (*R*^2^) suggested that not all subjects adapted their movements to the perturbation. Without motor adaptation, we could not hope for priming effects on learning. Indeed, comparing Bayesian and Akaike information criteria (BIC and AIC) between a “null” linear regression model that included only a constant term, and a model that included perturbation as an additional predictor for each individual, two subjects showed no evidence of motor adaptation to the perturbation (ΔBIC and/or ΔAIC in favor of the null model). Without these two subjects, the three-way interaction approached significance even when computed across all target locations [*t*(31) = 2.02, *P* = 0.052, d = 0.27]. Still, across target locations, the effect is substantially smaller than at the target location that displayed the largest initial movement variability, in line with high planning noise (i.e., the left target location; d = 0.7). To what extent factors of our study design, or limitations inherent to movement control, prevented us from observing significant priming effects across the workspace remains an open question for future research.

Our study lacks a condition that assesses baseline movement variability and baseline motor adaptation rate. Targets during priming had a highly task-relevant spatial dimension and a less task-relevant spatial dimension. As discussed, the present design cannot dissociate between potential priming effects on learning due to the high task-relevance of one dimension (and/or the consequent constraint on movement variability in that dimension) and potential priming effects due to the comparatively lower task relevance of the other dimension (and/or the consequent permission of movement variability). A full within-subject design that can dissociate between these possibilities would have included, ideally, four sessions; that is, adding to the present design a session in which priming involves “fully task-relevant” (e.g., small dot) targets and a session in which priming involves targets with low task-relevance in both spatial dimensions (e.g., large dots). As one of our reviewers pointed out, we can currently only conclude with certainty that variability and learning rate were scaled by our experimental manipulation.

Participants first completed the priming phase before learning were assessed in the subsequent test phase. An interleaved designed, in which learning is assessed in probe trials interspersed with priming trials, could, in principle, represent an alternative to our sequential design. However, frequently switching between priming trials (with arc- and line-shaped targets) and test trials (with dots as targets) may counteract the effect of priming on movement variability. For example, when priming trials are interleaved with test trials that require precision along both spatial dimensions, it is likely that participants try to maintain high precision even in priming trials, e.g., by moving toward the midpoint of arc- or line-shaped targets. Because of this potential concern, we chose a sequential design.

With this design, we provide the first evidence that workspace redundancy can modulate learning in one and the same individual, as compared with a largely nonredundant workspace, with potential implications for rehabilitation.

## DATA AVAILABILITY

Data will be made available upon reasonable request.

## SUPPLEMENTAL DATA

10.5281/zenodo.6980246 Supplemental Figs. S1 and S2: https://doi.org/10.5281/zenodo.6980246.

## GRANTS

M-P. Stenner was supported by a VolkswagenStiftung Freigeist Fellowship, project-ID 92977, and received funding from a Deutsche Forschungsgemeinschaft Sonderforschungsbereich, SFB-1436, TPC03, project-ID 425899996.

## DISCLOSURES

No conflict of interest, financial or otherwise, are declared by the authors.

## AUTHOR CONTRIBUTIONS

J.E., S.R., and M-P.S. conceived and designed research; J.E. and S.R. performed experiments; J.E., S.R., and M-P.S. analyzed data; J.E., S.R., and M-P.S. interpreted results of experiments; J.E., S.R., and M-P.S. prepared figures; J.E., S.R., and M-P.S. drafted manuscript; J.E., S.R., and M-P.S. edited and revised manuscript; J.E., S.R., and M-P.S. approved final version of manuscript.
